# Should the Right Coronary Artery Be Routinely Assessed During Provocative Spasm Testing?

**DOI:** 10.3390/jcm14041355

**Published:** 2025-02-18

**Authors:** Olivia Girolamo, Rosanna Tavella, David Di Fiore, Abdul Sheikh, Sivabaskari Pasupathy, Eng Lee Ooi, Jessica A. Marathe, Christopher Zeitz, John F. Beltrame

**Affiliations:** 1 Adelaide Medical School, University of Adelaide, Adelaide, SA 5005, Australia; olivia.girolamo@adelaide.edu.au (O.G.); rosanna.tavella@adelaide.edu.au (R.T.); drabdulrauf@gmail.com (A.S.); sivabaskari.pasupathy@adelaide.edu.au (S.P.); englee.ooi@adelaide.edu.au (E.L.O.); christopher.zeitz@sa.gov.au (C.Z.); 2 Basil Hetzel Institute for Translational Research, Woodville South, SA 5011, Australia; 3 Central Adelaide Local Health Network, Adelaide, SA 5000, Australia; 4 Albury Wodonga Health, Albury, NSW 2640, Australia; drdfiore@gmail.com; 5 Northern Adelaide Local Health Network, Adelaide, SA 5112, Australia; 6 Vascular Research Centre, South Australian Health and Medical Research Institute, Adelaide, SA 5000, Australia

**Keywords:** angina with non-obstructive coronary arteries, ANOCA, provocative spasm testing, coronary artery spasm, multivessel testing, functional coronary angiography

## Abstract

**Background/Objectives**: The diagnosis of coronary artery spasm (CAS) frequently requires invasive provocation testing, typically utilising acetylcholine (ACh). Although the left coronary artery (LCA) is routinely assessed as a part of the testing protocol, assessment of the right coronary artery (RCA) is often avoided since it requires the insertion of a temporary pacing wire. We sought to compare the prevalence of inducible CAS in the LCA and RCA, among patients with CAS undergoing multivessel spasm provocation testing with ACh. **Methods**: A local multi-institutional ANOCA (angina and non-obstructive coronary arteries) database was analysed, which included 316 patients with angina and suspected CAS who underwent provocation testing (single vessel *n* = 266, multivessel *n* = 50) with incremental bolus doses of intracoronary ACh (25, 50, 100 μg in the LCA; 25, 50 μg in the RCA). CAS was defined as >90% constriction of the epicardial coronary artery as assessed visually on coronary angiography. **Results**: In the 50 patients (55 ± 10 years, 77% female) who underwent multivessel spasm provocation testing, CAS was induced in 20 patients (40%), with ACh provoking CAS only in the LCA system in 45%, only in the RCA system in 35%, and both LCA/RCA in 20%. **Conclusions**: These findings demonstrate that assessing only the LCA may miss up to one-third of CAS cases. Therefore, it is essential to routinely evaluate the RCA, particularly when no inducible spasm is detected in the LCA.

## 1. Introduction

Angina is chest pain occurring due to inadequate coronary blood flow to the heart muscle. Typically, this arises from coronary artery stenoses that cause luminal narrowing, obstructing coronary blood flow; this can be readily visualised by coronary angiography. However, approximately one half of patients undergoing invasive coronary angiography for the investigation of angina symptoms, suspected to be of an ischaemic nature, are found to have non-obstructive coronary arteries (NOCA) [1]. These patients with angina and non-obstructive coronary arteries (ANOCA) represent a significant clinical challenge. Their diagnostic and treatment pathway falls outside the conventional approach and management of patients with obstructive coronary artery disease (CAD) due to atherosclerotic lesions. Patients with ANOCA are typically younger females with fewer cardiac risk factors and commonly present with recurrent, refractory angina and persistent symptoms [2]. ANOCA is associated with increased cardiovascular risk, high morbidity, an impaired quality of life and recurrent hospitalisations contributing to significant utilisation of health care resources and lost individual productivity [3]. As such, optimal care of patients with ANOCA is of the utmost importance. This is contingent upon accurate diagnosis and initiation of appropriate treatment that targets underlying pathophysiological mechanisms, typically involving coronary vasomotor dysfunction [4].

Coronary artery spasm is an important aetiology of ANOCA, accounting for a large proportion of diagnoses [5]. These patients characteristically experience sudden, severe chest pain, sometimes at rest, due to myocardial ischaemia arising from transient constriction of the coronary artery, impeding coronary blood flow [6]. The underlying pathophysiology of coronary artery spasm remains elusive, although there is a consensus that it is multifactorial. Coronary artery spasm involves mechanisms of endothelial dysfunction with impaired nitric oxide production and/or release, vascular smooth muscle cell hypersensitivity, and perivascular advential inflammation, all of which result in impaired vascular tone [6]. Anatomically, vasospasm may be diffuse or focal, occurring within a single vessel or multiple coronary arteries. Whilst the overall prognosis of coronary artery spasm is generally reported to be favourable [4], multivessel spasm is widely associated with increased cardiac risk and poorer patient outcomes [6,7].

Provocative spasm testing with acetylcholine (ACh) is considered to be the key test for diagnosing coronary artery spasm in patients with ANOCA [8]. ACh is an endothelium-dependent vasodilator. In normal physiological states, ACh acts on the muscarinic receptors of the coronary artery endothelium and vascular smooth muscle cells to induce vasodilation and increase blood flow to the myocardium. However, in patients with coronary artery spasm, characterised by endothelial dysfunction and impaired release of nitric oxide, ACh favours smooth muscle-mediated vasoconstriction [2]. Hence intracoronary ACh induces vasospasm in affected vessels, impeding coronary blood flow and causing angina symptoms [2].

The uptake of intracoronary provocative spasm testing has increased in cardiac catheterisation laboratories around the world, with growing recognition of ANOCA as a significant coronary syndrome [9]. Intracoronary ACh has been proven to be safe, with studies largely reporting a favourable safety profile of ACh provocative testing. There is relatively low procedure risk, and major complications reportedly occur at an incidence rate of 0.5–1.1% [9,10]. Provocative testing utilising intracoronary ACh has a high sensitivity (90%) and specificity (99%) [11], and as such is now indicated as a Class IB recommendation in managing ANOCA populations in recent international guidelines [12,13,14].

Provocative spasm testing protocols usually involve administering intracoronary ACh as a bolus dose into the left coronary artery, with observation of angiographic constriction within the epicardial coronary vessels ≥ 90% compared with the baseline considered diagnostic for inducible coronary artery spasm [2]. However, there is heterogeneity in expert opinion regarding routine testing of the right coronary artery. Whereas ACh administration into the left coronary artery rarely produces bradyarrhythmias, a bolus injection of ACh into the right coronary artery often produces a profound, transient, parasympathetic-mediated bradycardia requiring prophylactic temporary pacemaker insertion. The Japanese Circulation Society [15] recommends the insertion of a temporary pacing wire at the beginning of ACh provocation testing, with routine assessment of both the left and right coronary arteries. This not only ensures evaluation for right coronary artery spasm but also provides insights into the presence of multivessel spasm; an important predictor of poor outcomes in vasospastic angina [16]. In contrast, the Microvascular Network recommend ‘routine investigation of the left anterior descending artery only’ during provocative spasm testing [2]. This approach avoids the additional instrumentation and risk associated with temporary pacemaker insertion, but assumes that vasospasm is a ubiquitous phenomenon. This approach accepts that the presence/absence of spasm in the left coronary artery is sufficient for the assessment of coronary artery spasm. An intermediary approach is to test the left coronary artery first and proceed onto right coronary artery testing if no inducible left coronary artery spasm is observed.

The lack of a uniform global coronary artery spasm provocation protocol leads to variability in testing, and inevitably a degree of ambiguity in reported outcomes within the literature. A review of the literature over the last 10 years demonstrates this variability. For example, four key publications only conducted provocative testing in a single pre-specified target vessel [5,17,18,19], predominantly the left coronary artery/left anterior descending artery, and six only conducted multivessel provocative assessment to confirm single-vessel spasm (i.e., testing the right coronary artery if the left coronary artery is negative, or vice versa) [20,21,22,23,24,25].

To date, there has been limited investigation of the clinical importance of comprehensive multivessel provocative and functional testing to improve diagnostic accuracy in patients with ANOCA. The primary objective of this study is to assess the prevalence of ACh-induced left and right coronary artery epicardial spasm in patients with ANOCA and diagnosed with coronary artery spasm after undergoing multivessel provocative testing with intracoronary ACh.

## 2. Materials and Methods

This study employs a retrospective, observational analysis utilising a multi-institutional coronary spasm provocation testing database reflecting real-world clinical practise in Adelaide (Australia). In 316 patients who underwent ACh provocative testing during invasive coronary angiography between November 1995 and October 2023 (inclusive) across two tertiary sites, 50 patients underwent multivessel testing. Patients were included if they underwent elective invasive coronary angiography and were found to have symptomatic ANOCA, defined as the absence of coronary stenosis ≥ 50% in a major coronary artery. Additionally, the patients had experienced chest pain within the past month. Patients were excluded from the study if they had other cardiac causes of chest pain, such as obstructive coronary artery disease (coronary stenosis ≥ 50%), aortic stenosis, pulmonary embolism, pericarditis, or cardiomyopathy with an ejection fraction < 50%. Additional exclusion criteria included (1) a history of coronary artery bypass grafting surgery, (2) a clinical diagnosis of asthma, (3) the presence of a permanent pacemaker or defibrillator, (4) cognitive impairment that prevented obtaining informed consent, (5) inability to communicate in English, (6) being pregnant or at risk of pregnancy, and (7) admission for an acute coronary syndrome event in the preceding 3 months. Following routine diagnostic angiography, provocative spasm testing was conducted utilising a validated ACh provocative testing protocol [11]. The referring cardiologist granted permission for the temporary discontinuation of calcium channel blockers and/or nitrates for at least 24 h prior to the diagnostic angiography so as to not interfere with provocative testing results. All patients provided signed consent for this medication cessation. Intracoronary ACh was administered incrementally as a 20 s bolus into the left coronary artery (25, 50, and 100 μg) and right coronary artery (25 and 50 μg). The artery was imaged within 1 min of injection and there was a minimum 5 min interval between each ACh dose. Demonstration of spasm at lower doses precluded further ACh administration. For right coronary artery assessment, a temporary pacing lead was inserted into the right ventricle (with threshold set to 50 bpm). Provocative spasm testing was concluded following either the induction of occlusive/sub-occlusive epicardial spasm (≥90% constriction of a major epicardial coronary artery), or by attaining the maximal ACh dose without inducing coronary artery spasm. Intracoronary Glyceryl Trinitrate (GTN) was administered (150 μg bolus) to relieve induced coronary artery spasm, or at the end of the protocol to assess endothelium-independent function through observation coronary artery tone.

Although the ACh dosage administration remained consistent throughout the study period, the order of vessel assessment changed as this diagnostic investigation evolved. The early approach involved routine insertion of a temporary pacing wire, with the right coronary artery initially evaluated, followed by the left coronary artery if no spasm was induced. The rationale was that ACh-induced spasm was only required in one vessel for a diagnosis of coronary artery spasm and hence initiation of vasospastic therapy. In 2013, a comprehensive Functional Coronary Angiography protocol was developed at Central Adelaide Local Health Network. This involved deploying a combined dual-sensor tipped-pressure Doppler flow wire (ComboWire, Philips, CA, USA) into the left anterior descending artery to assess coronary microvascular function via adenosine-induced hyperaemia and thereafter performing ACh provocative spasm testing [26]. Consequently, ACh testing changed to assessing the left coronary artery first and if spasm was induced, the right coronary artery was not tested. However, if no left coronary artery spasm was observed, a temporary pacing wire was inserted, and the right coronary artery was tested. However, this practise was not adhered to by all operators, whereby only single-vessel testing was undertaken in either the left coronary artery or right coronary artery. All studies were conducted with monitoring of patient symptoms throughout the protocol, with the identification of reproducible angina with ACh provocation and continuous 12-lead ECG recordings for the evaluation of ischaemic changes in line with induced spasm. The study protocol was approved by the local human research ethics committee in line with the guidelines of the 1975 Declaration of Helsinki and informed consent for participating was obtained from all subjects involved in the study.

The primary endpoint of the study was to evaluate the effectiveness of the provocative testing approach at our institutions by determining the prevalence of coronary artery spasm in patients undergoing both single and multivessel testing and identifying the distribution of affected vessels.

Continuous variables were reported as the mean ± standard deviation (SD) and analysed using the Independent Samples *t*-test. Categorical variables were reported as n (%) and analysed using the Chi-squared test. *p*-values were calculated as two-tailed, with values < 0.05 considered statistically significant. Data analysis was performed using IBM SPSS Statistics, Version 23.

## 3. Results

In the 316 patients with ANOCA undergoing ACh provocative spasm testing (mean age of 55 ± 11 years, 78% females), 266 had only a single vessel tested, with 64% demonstrating inducible spasm (i.e., 47% in LCA, 17% in RCA) and thus not requiring additional testing for a diagnosis of coronary artery spasm. However, no inducible spasm was observed with single vessel testing in the remaining 96 patients, and thus a diagnosis of coronary artery spasm may have been missed. Importantly, 21% of these patients continued to experience chest pain symptoms, subsequently presenting to the Emergency Department within our health network over the next 12 months.

**Figure 1:**
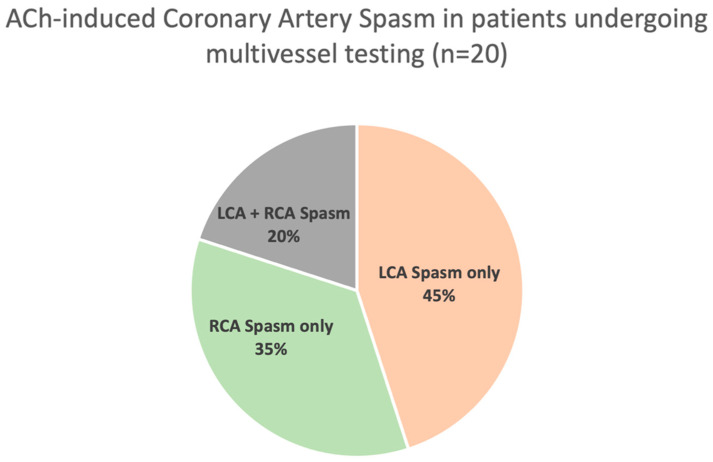
ACh-induced Coronary Artery Spasm in patients Undergoing Multivessel Testing (*n* = 20). Multivessel provocative testing was positive in 20 patients, 20% of which had spasm in both the left and right coronary arteries, 45% in the left coronary artery, and 35% in the right coronary artery alone. LCA: Left Coronary Artery, RCA: Right Coronary Artery.

Of the 50 patients (55 ± 10 years, 77% females) who underwent multivessel provocative testing, ACh-induced coronary artery spasm occurred in 20 patients (Figure 1). Amongst these 20 patients, ACh-induced spasm occurred in the left coronary artery only in 9 (45%), the right coronary artery only in 7 (35%), and both the left and right coronary arteries in 4 (20%). In four of seven patients with ACh-induced spasm in the right coronary artery, medical management was amended in line with first-line therapeutic recommendations with the use of calcium channel blockers—either increasing the continuing dose or commencing the medication. The remaining three patients had no change in their calcium channel blockers. Accordingly, if only the left coronary artery was assessed in these patients, then one third of patients would have potentially been incorrectly assumed to be negative for coronary artery spasm and would not have had optimised medical therapy. Extrapolating these findings to the above cohort of 96 patients, who were considered to be negative for coronary artery spasm after having no inducible spasm in single-vessel testing, potentially close to 30 patients may have been misdiagnosed and inappropriately managed.

Additionally, among the 316 patients tested, 186 patients (59%) had single-vessel spasm in either the left coronary artery or right coronary artery following single- or multivessel testing. Comparison of the clinical characteristics between these two patients groups demonstrated no statistically significant differences (Table 1). Therefore, this indicates that relying solely on clinical suspicion when undertaking a single versus multivessel provocative testing protocol might not be a prudent approach.

## 4. Discussion

This study quantitates the real-world experience of isolated ACh-induced right coronary artery spasm prevalence in patients undergoing multivessel provocative testing. The results demonstrate that as many as 1 in 3 patients may have a missed diagnosis if only left coronary artery provocative testing is undertaken. Additionally, comparative analysis of patients with single-vessel spasm in the left or right coronary artery shows no distinguishing factors that may guide clinical decision making regarding which vessel should be tested. Furthermore, 21% of patients who underwent single vessel testing where spasm was not induced with ACh provocation, and were therefore concluded to be negative for coronary artery spasm, continued to present to the hospital Emergency Department for chest pain symptoms within 12 months. This may implicate missed or inappropriate cardiac diagnosis due to an incomplete testing protocol.

These findings align with the results of a recent randomised control trial reported by Rehan et al. evaluating the diagnostic utility of multivessel Coronary Function Testing in patients with ANOCA [27]. This comprehensive, prospective analysis demonstrated that whilst coronary artery spasm was predominantly observed in the left coronary artery, isolated right coronary artery spasm occurred in 20% of patients, and that multivessel testing increased the diagnostic yield of coronary artery spasm by 12.5% (*p* = 0.004) [27]. Additionally, the authors report that isolated right coronary artery spasm inflicts a similar anginal burden (as measured by Seattle Angina Questionnaire responses) compared to isolated left coronary artery spasm, indicating the potential impact of misdiagnosis [27]. Furthermore, the study results demonstrate a trend toward greater angina burden in ANOCA patients with multivessel coronary artery spasm, where patients face a poorer angina-related quality of life, though this was not statistically significant [27]. This is in line with previous reports of the prognostic implications of multivessel coronary artery spasm. Among others, a model developed for risk stratification of coronary artery spasm patients in a large Japanese cohort by Takagi et al. indicated multivessel spasm as a significant predictor of major adverse cardiac events, particularly cardiac death and acute coronary syndrome events, as compared to patients with single-vessel spasm [6,7,17]. Whilst it is likely that there would be initiation of the appropriate therapy to address the underlying ischaemic cause of angina in ANOCA patients based on a positive diagnosis in single-vessel testing, there is a missed potential to consider the implications of multivessel spasm in this cohort. This is particularly important to delineate ongoing risk of major adverse cardiac event, previously been shown to have a multivariate hazard ratio of 1.69 (*p* = 0.039) [16], refractory angina, and reduced quality of life. The possible pathophysiological explanation for the observed clinical outcomes in patients with multivessel spasm relates to an increased propensity to severe myocardial ischaemia subsequent to more widespread disruption to coronary circulation [7]. Overall, the reports of Rehan et al. affirm our observational findings, and further solidify the hypothesis that solely testing the left coronary artery risks misdiagnosis of coronary artery spasm in patients with ANOCA. Such an approach may incorrectly identify patients as having ‘non-cardiac’ chest pain, which will inevitably impact patient outcomes.

The delayed and/or misdiagnosis of underlying ischaemic pathologies such as coronary artery spasm has major consequences for patients with ANOCA. These patients continue to face debilitating cardiac symptoms and poor quality of life, and may be subject to great ordeal in their pursuit of diagnostic clarity due to clinical shortcomings [28,29]. These clinical shortcomings, which extend beyond diagnostic uncertainty, have been previously described and are a major source of frustration for patients with ANOCA [28,29]. These patients are often subject to repeated invasive coronary angiograms and are at increased risk of major adverse cardiac events. As such, the precise identification of the underlying ANOCA mechanism is imperative for appropriate pharmacological management.

**Figure 2:**
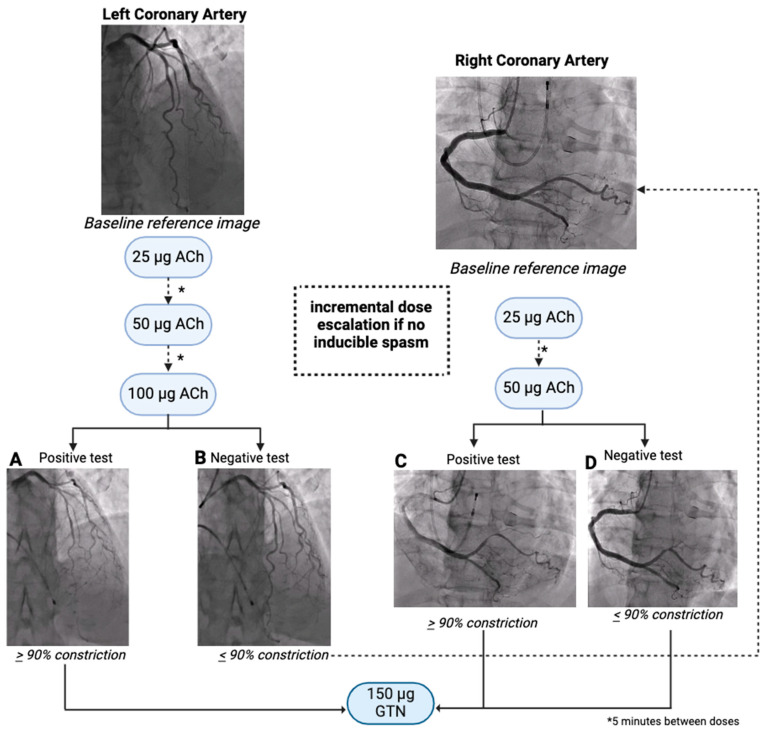
The workflow of a recommended provocative spasm testing protocol with intracoronary acetylcholine, at a minimum employing a multivessel testing approach in cases where single-vessel testing concludes a negative result. Following diagnostic invasive coronary angiography, whereby the patient is confirmed to have non-obstructive coronary arteries (atherosclerotic stenosis observed to be ≤50% vessel diameter, or a Fractional Flow Reserve ≥ 0.8 in any major epicardial coronary artery), provocative testing is initiated in the left coronary artery (LCA). An initial baseline image is used as a reference image before proceeding with an intracoronary acetylcholine (ACh) bolus dose of 25 μg over 20 s. A repeat angiogram image is taken to observe vasospasm, where <90% vasoconstriction is considered negative. Simultaneous monitoring of a 12-lead ECG for ischemic changes and patient-reported reproducible chest pain symptoms is conducted. The doses progress incrementally when negative (i.e., an increase of the dose to 50 μg when 25 μg does not result in vasospasm, and 100 μg when 50 μg does not result in vasospasm), * denotes the allowing of a 5 min interval between each dose before proceeding to the next step as necessary. LCA testing is concluded at a maximum dose of 100 μg or at 25 μg or 50 μg where occlusive or sub-occlusive vasospasm (>90% epicardial constriction) is induced (as in Image **A**). If LCA testing produces a negative result (as in Image **B**), RCA testing commences, demonstrated by the dashed arrow, following the insertion of a temporary pacing wire with the threshold set to 50 bpm, following the same pattern of progressive doses with negative results at 25 μg and 50 μg. RCA testing is concluded at a maximum dose of 50 μg, or at 25 μg where occlusive or sub-occlusive spasm is observed (as in Image **C**). If at the conclusion of this testing protocol RCA spasm produces a negative result (as in Image **D**), the patient is considered negative for coronary artery spasm. This concludes the administration of intracoronary ACh bolus doses, and is followed by a bolus dose of 150 μg intracoronary GTN to evaluate endothelium-independent coronary artery function, and/or to relieve ACh-induced coronary artery spasm and coinciding angina symptoms.

Our results demonstrate that there is regional variability in vasomotion dysfunction and that vasospasm is not a one-size-fits-all phenomenon; a negative result in one artery does not necessarily rule out spasm in the others. Thus, a comprehensive strategy is recommended, with routine provocative testing of the right coronary artery (with deployment of a prophylactic temporary pacing wire) being strongly indicated, particularly where the initial testing of the left coronary artery is negative, followed by GTN infusion for assessment of endothelium-independent function, as demonstrated in Figure 2. Failing to do so poses serious consequences for clinical decision making, including risk of missed diagnosis in the case of isolated single-vessel spasm in an untested vessel, or inappropriate diagnosis of single-vessel spasm in patients with a clinical history resembling potential multivessel spasm. ANOCA is generally considered to be a complex clinical phenotype which poses significant diagnostic challenges to clinicians. Implementation of a single-vessel provocative testing protocol perpetuates this notion, and introduces additional hindrance to the initiation of tailored treatment strategies which are crucial for optimal patient care.

### Study Limitations

This study is limited primarily in its retrospective and observational nature, and the relatively small sample size; particularly of the cohort who underwent multivessel testing. This restricts the potential generalisability of these findings to the broader ANOCA population. The sample size of 20 patients with a positive result after multivessel provocative testing lacks the statistical power needed to discern factors contributing to, or associated with, variability in diagnoses of different coronary artery affliction. Furthermore, this sample size was insufficient to definitively establish the clinical importance of multivessel testing in enhancing patient outcomes. Whilst this study has a small sample size over a long inclusion period, we report a provocative testing experience which spans over 20 years, and offer important insights given our extensive practise and understanding of the nuances and variability in these provocative protocols. As such, the findings of the study are evaluated with an in-depth appreciation of clinical relevance regarding appropriate patient diagnosis, management, and outcomes. Nonetheless, the study limitations warrant more widespread clinical recognition of the shortcomings of current diagnostic regimes for patients with ANOCA and coronary vasomotor dysfunction. The establishment of clinical groups, such as the COVADIS (Coronary Vasomotor Disorder International Study Group), the Microvascular Network (MVN), and the CSANZ (Cardiac Society of Australia and New Zealand) Coronary Vasomotor Working Group has played a key role in influencing and informing understanding of the importance of undertaking comprehensive coronary functional testing in patients with ANOCA. These groups pave the way towards promoting uniform protocols across the globe to improve management in this frequently overlooked cohort.

## 5. Conclusions

This study explicitly explores and documents the diagnostic yield of single versus multivessel ACh provocation testing for coronary artery spasm in patients with ANOCA in a real-world setting. The results demonstrate that epicardial coronary artery spasm may occur exclusively in the right coronary artery (i.e., in the absence of left coronary artery spasm) in up to one-third of patients. This indicates the risk of misdiagnosis in single-vessel testing targeting only the left coronary artery, and thus underscores the importance of undertaking a comprehensive assessment. Therefore, as a minimum standard, right coronary artery testing should be undertaken if left coronary artery spasm testing is negative. Although, some would advocate that multivessel testing should be routinely performed to assess for multivessel coronary artery spasm.

## Figures and Tables

**Table 1:** Clinical characteristics of patients with left coronary artery v right coronary artery single-vessel coronary artery spasm.

	LCA Spasm (*n* = 133)	RCA Spasm (*n* = 53)	*p*-Value
Age, years	55 (±12)	55 (±10)	0.937
Female sex	98 (72%)	37 (70%)	0.572
Smoking history	73 (55%)	27 (51%)	0.872
Hypertension	62 (47%)	31 (58%)	0.072
Diabetes	14 (11%)	10 (19%)	0.099
Dyslipidaemia	78 (59%)	29 (55%)	0.867

Values are mean ± SD or *n* (%); statistically significant difference *p* < 0.05. Comparison of clinical characteristics in patients with single-vessel spasm (*n* = 186) in either left coronary artery (*n* = 339) or right coronary artery (*n* = 53) demonstrating no significant difference between groups.

## Data Availability

The data presented in this study are available on reasonable request from the corresponding author.

## References

[1] Jespersen L., Hvelplund A., Abildstrøm S.Z., Pedersen F., Galatius S., Madsen J.K., Jørgensen E., Kelbæk H., Prescott E. (2012). Stable angina pectoris with no obstructive coronary artery disease is associated with increased risks of major adverse cardiovascular events. Eur. Heart J..

[2] Samuels B.A., Shah S.M., Widmer R.J., Kobayashi Y., Miner S.E.S., Taqueti V.R., Jeremias A., Albadri A., Blair J.A., Kearney K.E. (2023). Comprehensive Management of ANOCA, Part 1—Definition, Patient Population, and Diagnosis. J. Am. Coll. Cardiol..

[3] Tavella R., Cutri N., Tucker G., Adams R., Spertus J., Beltrame J.F. (2016). Natural history of patients with insignificant coronary artery disease. Eur. Heart J. Qual. Care Clin. Outcomes.

[4] Smilowitz N.R., Prasad M., Widmer R.J., Toleva O., Quesada O., Sutton N.R., Lerman A., Reynolds H.R., Kesarwani M., Savage M.P. (2023). Comprehensive Management of ANOCA, Part 2—Program Development, Treatment, and Research Initiatives. J. Am. Coll. Cardiol..

[5] Konst R.E., Damman P., Pellegrini D., Hartzema-Meijer M.J., van Uden B.J.C., Jansen T.P.J., Brandsma J., Vart P., Gehlmann H., Maas A. (2021). Vasomotor dysfunction in patients with angina and nonobstructive coronary artery disease is dominated by vasospasm. Int. J. Cardiol..

[6] Slavich M., Patel R.S. (2016). Coronary artery spasm: Current knowledge and residual uncertainties. Int. J. Cardiol. Heart Vasc..

[7] Han S.H., Lee K.Y., Her S.H., Ahn Y., Park K.-H., Kim D.-S., Yang T.-H., Choi D.-J., Suh J.-W., Kwon H.M. (2019). Impact of multi-vessel vasospastic angina on cardiovascular outcome. Atherosclerosis.

[8] Beltrame J.F., Crea F., Kaski J.C., Ogawa H., Ong P., Sechtem U., Shimokawa H., Bairey Merz C.N., Coronary Vasomotion Disorders International Study Group (2017). International standardization of diagnostic criteria for vasospastic angina. Eur. Heart J..

[9] Takahashi T., Samuels B.A., Li W., Parikh M.A., Wei J., Moses J.W., Fearon W.F., Henry T.D., Tremmel J.A., Kobayashi Y. (2022). Safety of Provocative Testing with Intracoronary Acetylcholine and Implications for Standard Protocols. J. Am. Coll. Cardiol..

[10] Ciliberti G., Seshasai S.R.K., Ambrosio G., Kaski J.C. (2017). Safety of intracoronary provocative testing for the diagnosis of coronary artery spasm. Int. J. Cardiol..

[11] Okumura K., Yasue H., Matsuyama K., Goto K., Miyagi H., Ogawa H., Matsuyama K. (1988). Sensitivity and specificity of intracoronary injection of acetylcholine for the induction of coronary artery spasm. J. Am. Coll. Cardiol..

[12] Gulati M., Levy P.D., Mukherjee D., Amsterdam E., Bhatt D.L., Birtcher K.K., Blankstein R., Boyd J., Bullock-Palmer R.P., Conejo T. (2021). 2021 AHA/ACC/ASE/CHEST/SAEM/SCCT/SCMR Guideline for the Evaluation and Diagnosis of Chest Pain: A Report of the American College of Cardiology/American Heart Association Joint Committee on Clinical Practice Guidelines. Circulation.

[13] Vrints C., Andreotti F., Koskinas K.C., Rossello X., Adamo M., Ainslie J., Banning A.P., Budaj A., Buechel R.R., Chiariello G.A. (2024). 2024 ESC Guidelines for the management of chronic coronary syndromes: Developed by the task force for the management of chronic coronary syndromes of the European Society of Cardiology (ESC) Endorsed by the European Association for Cardio-Thoracic Surgery (EACTS). Eur. Heart J..

[14] Kunadian V., Chieffo A., Camici P.G., Berry C., Escaned J., Maas A., Prescott E., Karam N., Appelman Y., Fraccaro C. (2021). An EAPCI Expert Consensus Document on Ischaemia with Non-Obstructive Coronary Arteries in Collaboration with European Society of Cardiology Working Group on Coronary Pathophysiology & Microcirculation Endorsed by Coronary Vasomotor Disorders International Study Group. EuroIntervention.

[15] JCS Joint Working Group (2014). Guidelines for Diagnosis and Treatment of Patients with Vasospastic Angina (Coronary Spastic Angina) (JCS 2013). Circ. J..

[16] Takagi Y., Takahashi J., Yasuda S., Miyata S., Tsunoda R., Ogata Y., Seki A., Sumiyoshi T., Matsui M., Goto T. (2013). Prognostic stratification of patients with vasospastic angina: A comprehensive clinical risk score developed by the Japanese Coronary Spasm Association. J. Am. Coll. Cardiol..

[17] Kim M.N., Kim H.L., Park S.M., Shin M.S., Yu C.W., Kim M.A., Hong K.S., Shim W.J. (2018). Association of epicardial adipose tissue with coronary spasm and coronary atherosclerosis in patients with chest pain: Analysis of data collated by the KoRean wOmen’S chest pain rEgistry (koROSE). Heart Vessel..

[18] Ford T.J., Stanley B., Good R., Rocchiccioli P., McEntegart M., Watkins S., Eteiba H., Shaukat A., Lindsay M., Robertson K. (2018). Stratified Medical Therapy Using Invasive Coronary Function Testing in Angina: The CorMicA Trial. J. Am. Coll. Cardiol..

[19] Jansen T.P.J., Konst R.E., de Vos A., Paradies V., Teerenstra S., van den Oord S.C.H., Dimitriu-Leen A., Maas A., Smits P.C., Damman P. (2022). Efficacy of Diltiazem to Improve Coronary Vasomotor Dysfunction in ANOCA: The EDIT-CMD Randomized Clinical Trial. JACC Cardiovasc. Imaging.

[20] Ong P., Athanasiadis A., Borgulya G., Vokshi I., Bastiaenen R., Kubik S., Hill S., Schaufele T., Mahrholdt H., Kaski J.C. (2014). Clinical usefulness, angiographic characteristics, and safety evaluation of intracoronary acetylcholine provocation testing among 921 consecutive white patients with unobstructed coronary arteries. Circulation.

[21] Ong P., Athanasiadis A., Hill S., Schaufele T., Mahrholdt H., Sechtem U. (2014). Coronary microvascular dysfunction assessed by intracoronary acetylcholine provocation testing is a frequent cause of ischemia and angina in patients with exercise-induced electrocardiographic changes and unobstructed coronary arteries. Clin. Cardiol..

[22] Hoshino M., Yonetsu T., Mizukami A., Matsuda Y., Yoshioka K., Sudo Y., Ninomiya R., Soeda M., Kuroda S., Ono M. (2016). Moderate vasomotor response to acetylcholine provocation test as an indicator of long-term prognosis. Heart Vessel..

[23] Schoenenberger A.W., Felber S., Gujer S., Moser A., Jamshidi P., Stuck A.E., Erne P. (2013). Invasive findings in patients with angina equivalent symptoms but no coronary artery disease; results from the heart quest cohort study. Int. J. Cardiol..

[24] Aziz A., Hansen H.S., Sechtem U., Prescott E., Ong P. (2017). Sex-Related Differences in Vasomotor Function in Patients with Angina and Unobstructed Coronary Arteries. J. Am. Coll. Cardiol..

[25] Seitz A., Gardezy J., Pirozzolo G., Probst S., Athanasiadis A., Hill S., Mahrholdt H., Bekeredjian R., Sechtem U., Ong P. (2020). Long-Term Follow-Up in Patients with Stable Angina and Unobstructed Coronary Arteries Undergoing Intracoronary Acetylcholine Testing. JACC Cardiovasc. Interv..

[26] Girolamo O., Tavella R., Zeitz C., Beltrame J. (2023). A 10-Year Experience in Functional Coronary Angiography. Heart Lung Circ..

[27] Rehan R., Wong C.C.Y., Weaver J., Chan W., Tremmel J.A., Fearon W.F., Ng M.K.C., Yong A.S.C. (2024). Multivessel Coronary Function Testing Increases Diagnostic Yield in Patients with Angina and Nonobstructive Coronary Arteries. JACC Cardiovasc. Interv..

[28] Tavella R., Beltrame J.F. (2022). Shortcomings in Managing Patients with Ischemia with Nonobstructed Coronary Arteries. Circ. Cardiovasc. Qual. Outcomes.

[29] Flanagan L. (2022). My Journey with Ischemia and Nonobstructed Coronary Arteries. Circ. Cardiovasc. Qual. Outcomes.

